# Pharmacogenomics and Personalized Medicine

**DOI:** 10.3390/genes11060679

**Published:** 2020-06-22

**Authors:** Erika Cecchin, Gabriele Stocco

**Affiliations:** 1 Experimental and Clinical Pharmacology, Centro di Riferimento Oncologico di Aviano (CRO) IRCCS, 33081 Aviano, Italy; 2 Department of Life Sciences, University of Trieste, 34127 Trieste, Italy

**Keywords:** pharmacogenomics, personalized medicine, human genetics, pharmacology

## Abstract

Pharmacogenomics is one of the emerging approaches to precision medicine, tailoring drug selection and dosing to the patient’s genetic features. In recent years, several pharmacogenetic guidelines have been published by international scientific consortia, but the uptake in clinical practice is still poor. Many coordinated international efforts are ongoing in order to overcome the existing barriers to pharmacogenomic implementation. On the other hand, existing validated pharmacogenomic markers can explain only a minor part of the observed clinical variability in the therapeutic outcome. New investigational approaches are warranted, including the study of the pharmacogenomic role of the immune system genetics and of previously neglected rare genetic variants, reported to account for a large part of the inter-individual variability in drug metabolism. In this Special Issue, we collected a series of articles covering many aspects of pharmacogenomics. These include clinical implementation of pharmacogenomics in clinical practice, development of tools or infrastractures to support this process, research of new pharmacogenomics markers to increase drug efficacy and safety, and the impact of rare genetic variants in pharmacogenomics.

Precision medicine has the ultimate goal of exactly matching each therapeutic intervention with the patient’s molecular profile. Over the last twenty years, the study of human genetics has been fueled by cutting-edge sequencing technologies leading to a deeper understanding of the relationship between genetic variation and human health [1]. The study of genetics has been widely applied in precision medicine, and one of the emerging applications is pharmacogenomics-informed pharmacotherapy, tailoring drug selection and dosing to the patient’s genetic features. To date, pharmacogenomic variation has an established role in drug efficacy and safety, enabling the creation of treatment guidelines by international scientific consortia aimed at creating medical guidance for the clinical application of pharmacogenomics. Specifically, the Clinical Pharmacogenetics Implementation Consortium (CPIC) and the Dutch Pharmacogenetics Working Group (DPWG) have developed validated guidelines for several drug-gene interactions that are made freely available as an on-line resource (www.pharmgkb.org) [2]. However, the uptake of pharmacogenomics into routine clinical care remains limited. A range of major barriers has been identified, spanning from basic pharmacogenomics research through implementation. The study of previously neglected rare genetic variants and the validation of their functional and clinical impact through the development of pre-clinical models and in silico tools is warranted to improve pharmacogenomic knowledge. On the other hand, ongoing international coordinated efforts set up to overcome the existing barriers to pharmacogenomic implementation will provide new tools and insights into the clinical application of pharmacogenomics, thus helping to pave the way for widespread adoption [3]. In this Special Issue, eleven papers are published, covering different aspects of research and clinical application in the field of pharmacogenomics.

Six papers report original results on the discovery of new genetic markers of the outcome of a pharmacological treatment in terms of either efficacy or toxicity. Two papers focus on the pharmacogenomics of platinum derivatives. Dugo and colleagues [4] report the results of the bioinformatic revision of a dataset of radically resected ovarian cancer patients from TCGA, treated with an adjuvant platinum-based treatment. They focus on tumor tissue genetic alterations and specifically on somatic copy number alteration, highlighting a significantly different pattern of genomic amplification in platinum resistant patients versus platinum sensitive. The paper underscores the importance of considering the tumor tissue genome when approaching the issue of pharmacogenomics in cancer treatment. Moreover, it points out the great opportunity offered by the large amount of genomic data produced by international consortia like TCGA that could be mined to highlight innovative pharmacogenomic markers. The research paper by Zazuli and colleagues [5] addresses the issue of predictive markers of nephrotoxicity due to cisplatin treatment. They attempt to validate some previously investigated genetic polymorphisms in *SLC22A2* and *ERCC2*. Quite interestingly, they aim to define whether different clinical definitions of nephrotoxicity (adjusted-AKI or CTCAE-AKI designation) could have contributed to previous inconsistent results on the predictive role of the analyzed variants. They report that the association with the polymorphisms was only significant when considering the nephrotoxicity definition according to CTCAE v4.03. This paper raises the important issue of the definitions of clinically relevant outcomes in pharmacogenomics, which may have hindered the generation of solid and reproducible data among various studies in the field. More generally, heterogeneity in ethnicity, demographic characteristics and treatment modalities (dose or co-treatment) could affect comparability among studies. Yanqui Xu and colleagues [6] describe an original analysis of publicly available data investigating effective drugs for breast cancer using a system approach. The analysis is focused on identifying molecules effective in particular breast cancer subtypes by considering the impact of potentially effective drugs on the pathway crosstalk mediated by miRNAs. In their integrated analysis, the authors point out, for example, sorafenib as a medication potentially effective on the basal subtype, or irinotecan for Her2-positive subtype. Al-Eitan and colleagues [7] evaluate the association between a panel of seven polymorphic variants in the well-established candidate genes *CYP2C9* (three variants) and *VKORC1* (four variants) and warfarin anticoagulant effects, in a cohort of unrelated Jordanian-Arab patients with cardiovascular disease. Warfarin response was evaluated in terms of the achievement of a coagulation level in the therapeutic range during therapy and of the drug dose required by the patient. Variants of both genes were associated with warfarin effects and dose requirement. Interestingly, the haplotype derived by the combination of the variants of each gene were also associated with the effects of warfarin, confirming the relevance of the multilocus *CYP2C9*/*VKORC1* genotype to improving warfarin therapy for Arab patients also. Lucafò and colleagues [8] evaluated the contribution of a panel of candidate genetic variants on the efficacy and pharmacokinetics and of azathioprine in a cohort of young Italian patients with inflammatory bowel disease. These variants included those well established in *TPMT*, but also in two highly polymorphic glutathione transferase enzymes, in particular the *GST-A1* and *GST-M1* isoforms. Interestingly, all variants affected azathioprine efficacy in this cohort. In particular, *TPMT* polymorphisms, associated with reduced enzymatic activity, determined improved response to azathioprine, due to reduced inactivation of the drug. On the other hand, variants determining reduced activity of *GST-A1* or *GST-M1* determined reduced azathioprine efficacy, likely because of a lower drug activation. The effect on azathioprine metabolite concentration and dose was confirmed for *GST-M1* and *TPMT*. Bise and colleagues [9] evaluate the potential involvement of miRNAs in determining the variation in expression levels of drug transporters or enzymes involved in the activation or inactivation of cytarabine and other analogs, an important mechanism potentially determining drug resistance. The authors evaluate miRNA and gene-expression levels of cytarabine metabolic pathway genes in 8 AML cell lines and the TCGA database, demonstrating that miR-34a-5p and miR-24-3p regulate DCK, an enzyme involved in activation of cytarabine, and DCTD, an enzyme involved in metabolic inactivation of cytarabine expression, respectively. The authors also confirmed the binding of these mRNA–miRNA pairs on the basis of gel shift assays.

Three papers report the results of research work aimed at investigating how to improve pharmacogenomic implementation in clinical practice. The work of Lunenburg and colleagues [10] approached the theme of rare genetic profiles that are not included in the current version of pharmacogenomic guidelines, and the importance of integrating phenotyping strategies into genotyping in these cases. Specifically, they investigated seven cases of rare occurrence of *DPYD* compound heterozygosity for two of the four *DPYD* genetic polymorphisms with a validated effect on fluoropyrimidines safety. The most difficult task in these cases is the phasing of the genotypes in order to obtain a proper translation of genotype to phenotype. Since currently available phasing strategies are difficult to translate into a diagnostic routine, the authors point out the necessity in these sporadic cases of performing DPD phenotyping based on the measurement of DPD activity, in order to define the real enzymatic capacity of each individual. The paper by Roncato et al. [11] describes the development of FARMAPRICE, an IT-based clinical decision support system (CDSS) for the user-friendly application of existing pharmacogenomic guidelines in the clinical practice of drug prescription in Italy. The lack of dedicated IT tools is an acknowledged barrier to the implementation of pharmacogenomics. Even if the usability of electronic health records must be greatly improved in order to allow an effective translation of genetic information into routine drug prescription in Italy, the development of tools like FARMAPRICE can be helpful in facilitating the process. Another paper by Van Der Wouden and colleagues [12] investigated the up-take of a similar tool in a different European health care system. A pharmacogenomic CDSS is currently in use in the Netherlands and is fully integrated with patients’ electronic health records. Specifically, the study reports the results of the uptake of this tool within a prospective pilot study with community pharmacies (the Implementation of Pharmacogenetics into Primary care Project (IP3) study). Two hundred patients were pre-emptively genotyped for eight pharmacogenes, and the genotypes were embedded in the electronic health records. The data were used by pharmacists and general practitioners for the purposes of drug prescription. The approach was demonstrated to be feasible in the context of primary care and manageable for pharmacists and general practitioners. Almost all of the patients had the opportunity to re-use their genetic data more than once and about one fourth of the patients had at least one actionable piece of information in their pharmacogenetic passport.

This special issue also includes two outstanding literature reviews. Davila-Fajardo’s [13] revision is focused on implementation/cardiology. Indeed, drugs used in this clinical setting have a huge interindividual variability, which is reflected in highly impactful under- or over-treatment, which severely affects the safety of the patients. The choice of the drug and the dose is often critical, and strict clinical monitoring is required to adjust the treatment, as in the case of warfarin. Many gene–drug interactions are available that have been validated by large prospective clinical trials with the opportunity to integrate clinical and genetic information in predictive pharmacogenetic algorithms. Cost-effectiveness studies were also conducted supporting the application of PGx information in the dose adjustment. In conclusion, PGx tests for clopidogrel in high-risk patients and warfarin in patients including all indications could begin to be implemented in daily clinical practice, similar to simvastatin tests. Acenocoumarol should be limited to patients who do not reach the INR after a certain period of treatment. The algorithm could improve acenocoumarol dosage selection for patients who will begin treatment with this drug, especially in extreme-dosage patients. Further studies are necessary to confirm that the PGx test for acenocoumarol is ready for use. Pavlovic and colleagues [14] summarized the contribution of high-throughput technologies, including microarrays and next-generation sequencing, to the pharmacogenomics and pharmacotranscriptomics of pediatric acute lymphoblastic leukemia (ALL). Emerging molecular markers responsible for the efficacy, adverse effects and toxicity of the drugs commonly used for pediatric ALL therapy, i.e., glucocorticoids, vinka alkaloids, asparaginase, anthracyclines, thiopurines and methotrexate are presented in the review. For instance, among the most promising, the authors describe *CEP72* rs924607 TT genotype and its association with vincristine induced neuropathy. The authors underline that while a significant amount of data has been generated using high-throughput technologies, the clinical implementation of these findings is still limited. To increase clinical implementation of this outstanding research, the authors discuss the relevance of data analysis and of designing prediction models using bioinformatics, machine learning algorithms and artificial intelligence.

In conclusion, the studies collected in this volume underline the potential of innovative molecular approaches, including multilocus genotyping, sequencing of rare variants and epigenetic features, in identifying genetic determinants of interindividual variability in the effects of drugs in several important clinical settings, including chemotherapy of breast cancer and leukemia and anticoagulant therapy for cardiovascular diseases. The integration of multiple layers of pharmacological information, including variation in gene expression and function of drug targets, pharmacokinetic profiles, also obtained through innovative statistical and bioinformatic approaches, holds the potential of explaining the predictable sources of interpatient variability in drug effects, which properly implemented will bring to precision therapy.
